# A new method for lattice reduction using directional and hyperplanar shearing

**DOI:** 10.1107/S2053273321011037

**Published:** 2022-01-01

**Authors:** Cyril Cayron

**Affiliations:** a Laboratory of Thermo Mechanical Metallurgy (LMTM), PX Group Chair, EPFL, Rue de la Maladière 71b, Neuchâtel, 2000, Switzerland

**Keywords:** lattice reduction, hyperplane, left inverse, algorithm

## Abstract

A geometric method of lattice reduction based on cycles of directional and hyperplanar shears is presented. The deviation from cubicity at each step of the reduction is evaluated by a parameter called ‘basis rhombicity’ which is the sum of the absolute values of the elements of the metric tensor associated with the basis. The levels of reduction are quite similar to those obtained with the Lenstra–Lenstra–Lovász (LLL) algorithm, at least up to the moderate dimensions that have been tested (lower than 20). The method can be used to reduce unit cells attached to given hyperplanes.

## Abstract 2

A new algorithm for lattice reduction based on a series of directional and hyperplanar shears and driven by the decrease of the basis rhombicity is proposed. It can be used to reduce unit cells in dimension 3 and higher.

## 1. Introduction

In a recent paper (Cayron, 2021 ▸), we proposed a method to determine a unit cell attached to any hyperplane 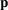 . A hyperplane 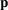 is a plane of dimension 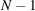 in a space of dimension *N*. Its Miller indices 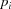 permit it to be built geometrically in the direct basis by considering its intersection points with the *i*th axes (in 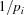 ). Equivalently the letter 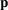 represents the vector of coordinates 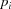 in the reciprocal basis; this vector is normal to the hyperplane. The unit cell attached to the hyperplane 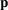 is made of one short vector 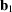 pointing to a node of the first layer parallel to the plane 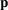 , *i.e.* such that the scalar product 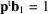 , and of 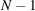 short vectors 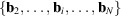 lying in the plane 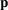 , *i.e.* such that the scalar product 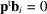 , where ‘t’ means ‘transpose’. The first vector is a solution of Bézout’s identity, and the 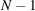 vectors are solutions of the integer relation, both with the coordinates 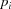 . Even if the vectors 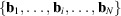 determined by the algorithm are already quite short, they can be reduced even more, *i.e.* it is possible to find shorter vectors 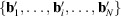 defining a smaller and more orthogonal unit cell of the same volume associated with the same hyperplane 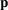 , *i.e.* fulfilling the same Bézout’s identity and integer relation. Reducing the length of the vectors in a lattice is related to the general problem called ‘lattice reduction’.

Let us explain it in a general way. Given a lattice 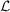 spanned (freely) by *N* vectors 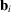 , lattice reduction consists of finding new relatively short, nearly orthogonal vectors 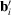 spanning the same lattice 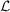 . The reduced and initial bases are linked by integers 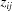 such that 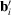 = 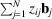 and 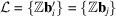 , where the { 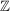 } means all linear combinations with integer coefficients. The number of vectors cannot be larger than the space dimension. The coefficients 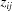 form a unimodular matrix 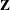 (integer matrix of determinant ±1), and the relation between the vectors of the bases is

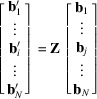

with 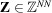 and 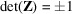 , where 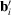 and 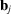 refer to the vectors themselves, not to their coordinates. Strictly speaking, it is the basis whose vectors generate the lattice that is reduced at constant volume, and not the lattice itself since this remains the same. The most popular algorithm to determine a reduced basis is the Lenstra–Lenstra–Lovász (LLL) algorithm, which relies on Gram–Schmidt orthogonalization (Appendix *A* [App appa]). It is usual in lattice reduction problems to present the vectors 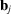 as the rows of a matrix. In crystallography, we generally write the coordinates in columns and keep the row notation for planes, *i.e.* for vectors of the reciprocal space. In order to avoid any confusion, we will write 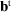 for a row vector 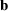 . With this notation, in a space of dimension *N*, a vector 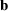 is an 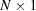 matrix, and 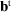 a 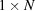 matrix. All the vectors in this paper are written in a Cartesian orthonormal basis and their coordinates are integers. The relation between the reduced and initial bases can be written in the form of a matrix product 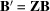 , where 

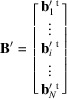

and 

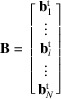

are matrices of 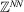 . A typical low-dimensional example of lattice reduction is the set of three vectors in three dimensions, 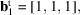 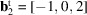 and 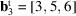 . They form the matrix

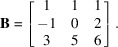

The reduced lattice basis is 

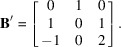

One can check that the three row vectors are 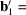 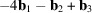 , 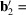 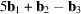 and 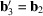 . The integer coefficients of linearity could be found by calculating 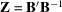 .

A direct lattice reduction algorithm, such as LLL, permits the lattice to be reduced but does not preserve the unit cell attached to a given hyperplane 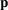 . We are thus looking for an intermediate method such that the vector 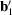 continues to point towards a node of the first layer, and the other vectors 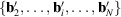 remain in the hyperplane 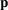 . An intuitive solution to reduce 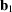 consists of applying a simple shear parallel to the hyperplane 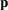 , as illustrated in Fig. 4 of Cayron (2021 ▸). One could then think of applying the LLL algorithm to reduce the other vectors 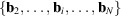 lying in the hyperplane 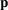 , but it is also possible to reverse the problem and use the intermediate simple shear method to develop a simple geometric algorithm of lattice reduction. This method, which we call ‘cubification’, is different from LLL because it does not require Gram–Schmidt orthogonalization. It is also well adapted to determine a reduced unit cell attached to a hyperplane 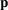 , as shown by Cayron (2021 ▸). It consists of applying simple shears parallel to the directions and to the hyperplanes of the lattice. Here, the term ‘shear’ should be understood in its general meaning: for a vector space 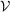 and a subspace 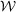 , a shear of a vector 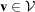 fixing 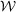 translates 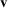 in a direction parallel to 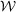 . If 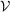 is the direct sum 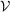 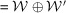 , we write 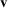 = **w** + **w**′, then the image of 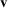 by the shear *S* is 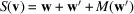 where *M* is a linear mapping from 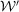 onto 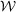 . Directional and hyperplanar shears correspond to the case where the dimensions of the subspaces 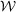 are 1 and 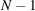 , respectively.

In general, a parameter called ‘orthogonality defect’ is used to evaluate the degree of reduction. It is defined by 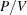 where *P* is the product of the norms of the basis vectors, 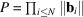 , and *V* is the volume of the cell formed by the vectors, 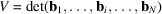 , which is an invariant of the reduction process. Another parameter to evaluate the norms could be 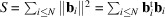 . In this paper, instead of using 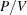 , the degree of ‘cubicity’ of a basis 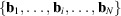 will be evaluated by calculating the ‘basis rhombicity’ defined from the Euclidean scalar products between the vectors:

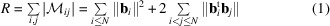

where 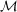 is the metric tensor given

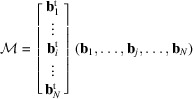




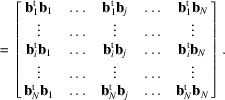




The ‘basis rhombicity’ contains the information on both the norms and the angles between the vectors. A lowest ‘rhombicity’ indicates a more cubic cell. Note that the term ‘rhombicity’ has a specific meaning in a branch of mathematics that deals with symmetric second-rank tensors in three-dimensional Euclidean space, but that is not the one given in the present paper. The ‘basis rhombicity’ *R* was preferred to the parameter *P*/*V* for two reasons:

(*a*) From a theoretical point of view, although it seems to be common knowledge, we realized that minimizing the norms of the vectors in high dimensions is not exactly equivalent to improving the orthogonality between them. The reader can look at the simple example in dimension 4 in Appendix *B* [App appb], which presents two bases of the same lattice, with the same norms *S* and *P*, but one with a better orthogonality, *i.e.* lower *R*, than the other one. We will also show in Section 4.3 an example in dimension 20 in which, for the same lattice, one reduced basis has a better orthogonality (lower *R*) but a worse norm (larger *P* and *S*) than another reduced basis.

(*b*) From a practical point of view, we noticed that the ‘cubification’ method leads to lower norms in terms of *P* or *S* when *R* is used as driving criterion, and not *P* or *S* themselves.

**Figure 1:**
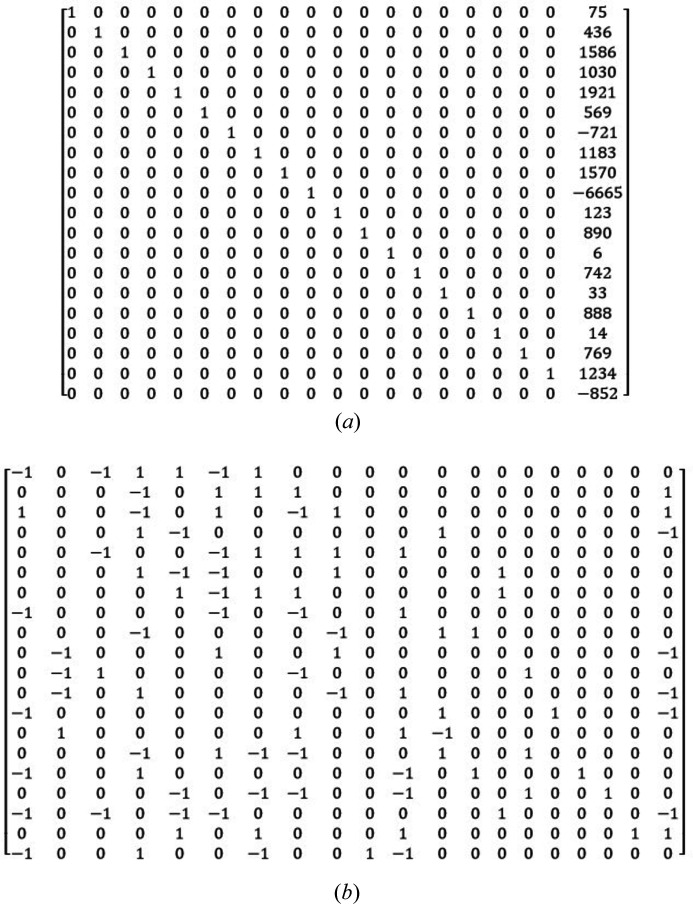
Example of the LLL algorithm with a 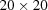 matrix representing a list of 20 vectors whose coordinates are written in rows. (*a*) Input list. (*b*) Output list determined with the function *LatticeReduce* of *Mathe­matica*. (*a*) Basis before reduction; the values of the rhombicity 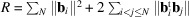 and of the sum of the squares of the norms 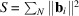 are *R* = 453988268, *S* = 61580172. (*b*) Basis after reduction; the parameters decreased to *R* = 531, *S* = 99.

Fig. 1 ▸(*a*) gives an example of lattice reduction with the LLL algorithm. The matrix representing the basis to be reduced is nearly the identity except that the last column containing the *N*th coordinates of the vectors is constituted of relatively high integers. These types of matrices are often used because they appear in the ‘knapsack problems’ (given a set of items, each with a weight and a value, one has to determine the number of each item to include in the knapsack so that the total weight should not exceed a limit and the value is maximized). Geometrically, the initial basis is highly elongated along the *N*th axis. Its initial values are *R* = 453988268, *S* = 61580172. They decrease to *R* = 531, *S* = 99 with the LLL-reduced basis given in Fig. 1 ▸(*b*).

The principle of directional shear will be presented in Section 2. It helps to obtain a reduced lattice with significantly lower *R* and *S* values, although higher than with LLL. The hyperplanar shear will be explained in Section 3; it permits *R* and *S* to be decreased further. In Section 4, it will be shown how cycling directional and hyperplanar shears permits values of *R* and *S* to be obtained that are comparable with those of LLL.

## 2. Directional shearing

### 2.1. Lagrange’s division

**Figure 2:**
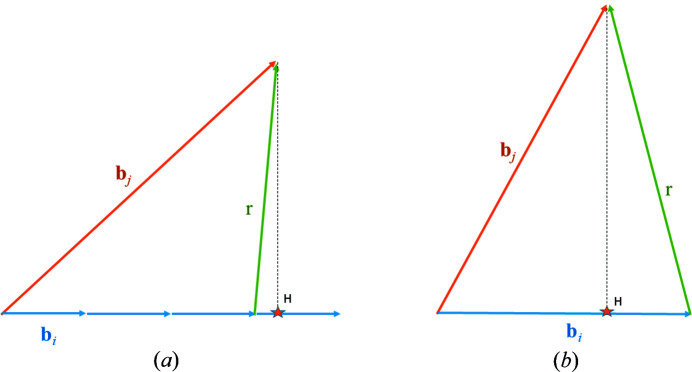
Directional shear of 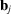 along the direction 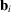 . Case where (*a*) 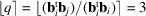 , and (*b*) 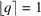 . The orthogonal projection point is noted *H* and marked by a little orange star.

Let us consider two vectors 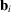 and 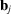 such that 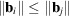 . We introduce the rational number 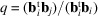 from the orthogonal projection of 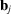 on 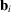 (Fig. 2 ▸). Practically, as in LLL, *q* is encoded by a floating-point number. The vector 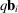 is rational and can be approximated by the integer vector 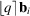 , where 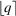 is the integer closest to *q* computed by 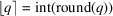 . The reduced vector 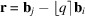 belongs to the lattice spanned by 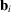 and 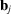 , and its norm is such that 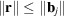 if the coordinates of 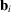 and 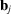 are such that 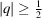 , *i.e.* 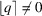 . In the limit case 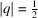 , the triangle made by ( 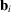 , 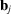 , 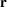 ) is isosceles, *i.e.* 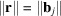 . Note that, in some cases, the norm of 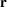 that is lower than that of 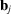 may even be lower than that of 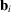 . The vector 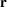 can be considered as the remainder of the vector division of 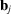 by 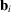 .

Now, we consider a basis in *N* dimensions made of *N* integer vectors 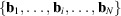 initially sorted by norms, from the lowest to the highest norms, *i.e.* such that 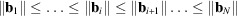 . The function ‘Lagrange’s division’ consists of applying vector divisions to the pairs of vectors 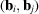 of the list. It starts with the vectors 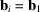 and 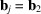 . Two cases should be distinguished in the algorithm: if 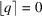 , nothing changes in the list and the next pair of vectors 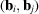 is considered by iteration with a loop with *i* containing a loop with *j*; and if 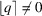 , the list is modified, and two algorithm variants are proposed:

Variant *Append*: the vectors 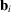 and 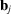 are deleted from the list, and the vectors 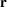 and 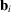 are appended at the end of the list.

Variant *Insert*: if 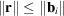 , 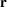 replaces 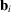 , and 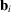 replaces 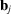 in the list; else, 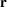 replaces 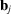 in the list.

The process is repeated recursively; the input for the function ‘Lagrange’s division’ is the new list of vectors (without sorting them). The recursion stops when all the values 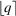 become null for all the pairs of vectors in the basis. The method is quite similar to Lagrange’s division described by Nguyen & Vallée (2010 ▸).

The variant *Insert* gives good results in a short time. The rhombicity and the sum of the squares of the norms of the list in Fig. 1 ▸(*a*) that were initially *R* = 453988268, *S* = 61580172 are reduced to *R* = 540, *S* = 134. These values are not far from those obtained with the LLL algorithm (*R* = 531, *S* = 99). With *Append*, the list of Fig. 1 ▸(*a*) is reduced ‘only’ to *R* = 1199, *S* = 337, but, as will be shown in the next sections, this will leave more action for the hyperplanar shearing, and better final reduction will be obtained at the end of the process for dimensions approximately 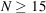 .

### 2.2. Simplification

Lagrange’s division reduces the vectors by pairs without considering the basis as a whole. Now, if one accepts to slightly but only temporarily degrade the value of *S* of the basis, the rhombicity *R* can be further improved as follows. Let us consider again a list of integer vectors 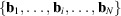 sorted by norms from the lowest to the highest norms. For a pair of vectors 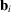 and 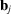 in the list such that 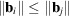 , we calculate the vector 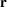 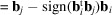 , where 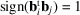 if 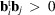 , 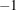 if 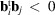 and 0 if 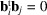 . Then, we calculate whether or not replacing 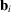 or 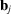 by 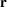 allows the value of the rhombicity *R* to be decreased. If the answer is positive, the change is made. Here again, two algorithm variants are proposed

Variant ‘*Append*’: if replacing 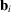 by 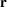 allows the value of *R* to be decreased, the vector 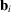 is deleted and the vector 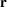 is appended at the end of the list. If not, the vector 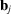 is deleted and the vector 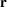 is appended at the end of the list.

Variant ‘*Insert*’: if replacing 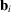 by 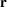 allows the value of *R* to be decreased, the vector 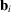 is replaced by 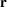 at its position *i*; else, 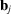 is replaced by 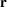 at its position *j*. The new list of vectors is then sorted again following the increasing norms.

The variant ‘*Insert*’ is chosen by default, except for random matrices for which the variant ‘*Append*’ should be preferred, as will be discussed in Section 4. The process of simplification is repeated recursively until *R* cannot be reduced anymore. Simplification permits the values obtained in Section 2.1 to be decreased a little more. For the list of Fig. 1 ▸(*a*), from the lattice reduced by Lagrange’s division with *R* = 1199, *S* = 337, the lattice is further reduced to *R* = 1084, *S* = 330 by simplification with the variant *Insert*. At this step, the rhombicity cannot be further reduced, even by combining Lagrange’s division and simplification. In the rest of the paper, the process described in Section 2 will be called ‘directional shearing’.

## 3. Hyperplanar shearing

### 3.1. The hyperplane normal

Let us consider again a list of integer vectors 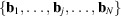 initially sorted by norms, *i.e.* such that 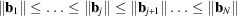 . We isolate the first vector 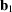 and the subspace of dimension 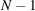 (hyperplane) constituted by the vectors 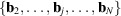 . The coordinates of the integer vector 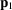 that is normal to this hyperplane can be calculated as follows. We write the coordinates of vectors 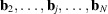 in columns to form the 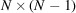 matrix

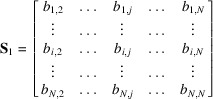

where 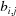 means the *i*th coordinate of the vector 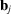 .

If we insert in the matrix a first column made of any vector of the set 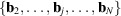 , let us say the vector 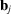 , then the new set of vectors becomes linearly dependent and the determinant of the *N* × *N* matrix is null:

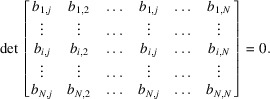




Let us write this determinant by its cofactor expansion along the first column. The minors, *i.e.* the determinants of 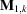 , the 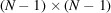 submatrices of 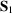 obtained by deleting the *k*th row, form a vector 

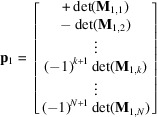

that fulfils the property 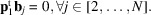 In other words, 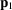 is the normal to the hyperplane 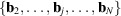 that we were looking for. Its norm equals the area of the hypersurface formed by the vectors 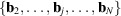 . The reader can check that in three dimensions 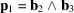 . The coordinates of 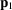 are the Miller indices.

*Note 1*. The calculation of the coordinates of 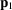 from the determinants of the square matrices 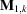 may appear complicated and computationally expensive, and one may think about other methods. It can be noticed that the coordinates of 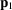 are the solution of 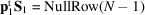 , the null row vector, or equivalently 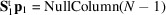 , the null column vector, both of dimension 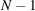 . This system of equations is underdetermined since it is constituted of 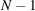 equations with *N* unknown. It can be solved by matrix inversion by imposing a specific value 0 or 1 to one of the coordinates of 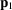 , but such an approach becomes numerically unstable and leads to incorrect solutions in high dimensions 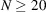 . A more classical way would be to compute Gaussian elimination taking care with the choices of the pivot positions to avoid instabilities, but the complexity is 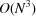 , which is comparable with that required to calculate *N* determinants of square matrices of dimension 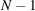 .

### 3.2. Hyperplanar shear

**Figure 3:**
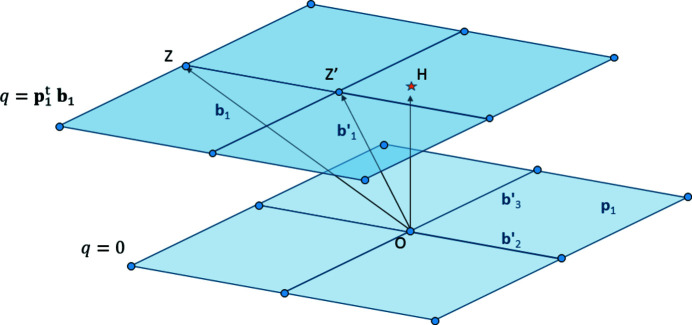
Hyperplanar shear parallel to 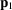 . The lattice is ‘stratified’ into different layers parallel to 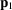 . The layer to which the vector 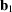 points is given by the integer 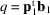 . The hyperplanar shear is made by calculating the point *H* (marked by a little orange star) which is the orthogonal projection of the origin *O* onto the layer *q*. The node *Z* such that 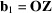 can be translated towards another node *Z*′ closer to *H* (see text). The vector 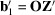 has a lower norm and a better ‘orthogonality’ with the hyperplane 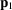 .

Let us consider a cell of the lattice 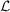 attached to the hyperplane 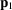 generated by the vectors 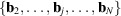 , *i.e.* 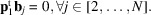 There are many equivalent cells, but we are looking for a quasi-reduced one. First, we replace the sublattice 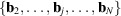 by its reduced form 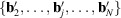 obtained by directional shearing, as described in Section 2. If this reduction in dimension 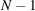 is not possible, the sublattice 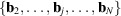 is not changed, *i.e.* 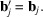 All the vectors 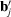 belong to the hyperplane 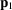 ; we say that they are in the layer *q* = 0 of the plane 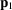 . Only the vector 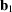 points to a node of the layer *q* of the hyperplane 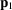 with 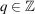 and 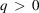 . Note that *q* = 1 for a unit cell. The set 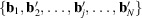 is a cell attached to the hyperplane (Cayron, 2021 ▸). Another vector of the lattice 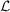 pointing to the layer *q* such as 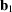 but shorter than 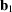 can be determined as follows. We note *O* the origin of the lattice, and *Z* the point such that 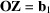 , as illustrated in Fig. 3 ▸. We call *H* the orthogonal projection of *O* on the layer *q* of the hyperplane 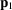 . It is such that 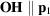 and 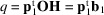 . Thus, 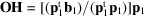 . Its coordinates are not integer but remain rational.

The vector 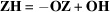 is a vector of the hyperplane 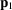 , which means that it can be written as a linear combination of the vectors 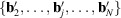 . In order to get its coordinates, we use again the 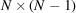 matrix formed by writing the reduced vectors in columns, *i.e.*


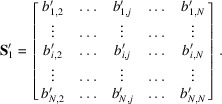

The 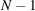 local coordinates of 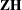 in the basis 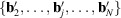 are given by 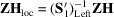 where 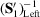 is the left inverse of the matrix 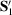 . We recall that a left inverse of a non-square matrix 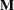 is 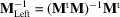 . The vector 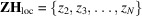 is an 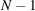 -dimensional rational vector in the 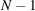 subspace. A lattice point Z′ close to *H* that belongs to the same layer is given by 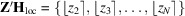 . The vector 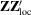 = 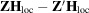 is calculated and re-expressed in the *N*-dimensional space by 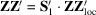 . The vector 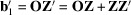 is a reduced form of the vector 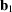 . At this step the cell 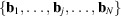 attached to the hyperplane 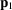 has been reduced; the new vectors defining this cell are 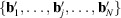 . This is the method used by Cayron (2021 ▸).

*Note 2*. The calculation of the 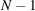 local coordinates of 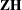 in the basis 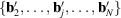 from the 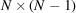 matrix 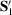 may appear complicated and computationally expensive, and one may think about other methods. One may notice that the coordinates of 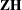 form an 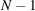 vector 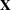 that is the solution of 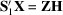 . The system of equations is overdetermined since it is constituted of *N* equations with 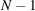 unknown (the coordinates of 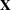 ). One could ignore one of the equations (*i.e.* remove one of the rows of 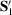 ) to solve the system by matrix inversion, but such an approach becomes numerically unstable and leads to incorrect solutions for high dimension 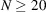 . This problem is induced by the projection. Let us explain it with an arbitrary example in three dimensions. We consider 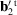 = [1211, 1423, 1] and 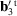 = [−8921, 2389, 1], two vectors nearly perpendicular to the *z* axis, and the vector 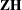 that is in the plane ( 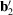 , 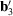 ). If we work with the coordinates (*x*, *y*) of 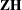 to write it as a linear combination of 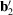 and 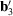 , a solution is found without any problem. However, if the coordinates (*x*, *z*) or (*y*, *z*) of 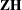 are used, then the system becomes ‘unbalanced’, and it would become completely unsolvable if 0 were chosen in place of 1 for the *z* coordinates of the vectors 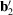 and 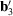 . Geometrically, the instability comes from the projection along a direction that makes the rhombus ( 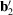 , 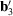 ) appear nearly on its edge, as a segment. To avoid this problem, one could solve the overdetermined system by Gaussian elimination, taking care with the choices of the pivot positions to avoid instabilities, but the complexity would be comparable with that required to calculate the left inverses of matrices.

The function ‘hyperplanar shear’ works as follows. It starts with the list 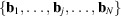 and it tries to reduce 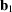 by a shear on the hyperplane 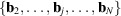 , as described previously. If the basis rhombicity is reduced when 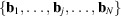 is substituted by 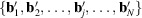 , the vector 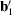 is moved to the end of the list, and the function is called again with 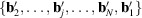 as input. If the rhombicity is not reduced, the function keeps the initial list 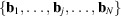 and tries to reduce the vector 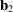 by a shear on the hyperplane 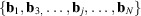 *etc*. The process stops when all the vectors 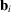 of the list 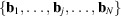 are screened but none of the vectors 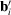 permits the basis rhombicity to be reduced any further. This series of hyperplanar shears will be called ‘hyperplanar shearing’.

Both directional and hyperplanar shearing imply orthogonal projections followed by numerical rounding in which rational numbers are replaced by their closest integers, which is actually very similar to the operations required in the Gram–Schmidt procedure. The lattice of Fig. 1 ▸(*a*) that was previously reduced by directional shearing becomes even more reduced by hyperplanar shearing: the rhombicity and sum of the squares of the norms decreased to *R* = 451 and *S* = 113. These values are closer to those obtained by LLL, and they will be improved even more by alternating directional and hyperplanar shearing, as detailed in the next section.

## 4. Cycling directional and hyperplanar shearing

### 4.1. Methods and options

The directional and hyperplanar shearing steps can now be repeated in cycles until the rhombicity cannot be decreased anymore. This method is called ‘cubification’. There is not a unique way to perform a cubification as it can be started by the directional shearing or by the hyperplanar shearing. It also depends on the variant of the algorithms chosen for Lagrange’s division (Section 2.1) and for the simplification (Section 2.2). By trial and error, we could identify two cubification methods (Table 1 ▸).

The chosen algorithm variant depends on the type of matrix that is to be reduced (Table 2 ▸). We refer to ‘columnar matrix’ as a list of vectors whose matrix (the vectors are written in rows) contains many zeros, and at least one column contains many non-null and generally moderate integer values (here 3 or 4 digits). A typical example is the matrix given in Fig. 1 ▸(*a*). We noticed that for matrices of dimensions approximately 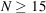 , Lagrange’s division in its *Append* variant gives better results than with ‘*Insert*’. A ‘heterogeneous matrix’ is a matrix that contains many zeros, and at least one row and one column with many non-null and moderate integer values. We noticed that for some cases of large heterogeneous matrices, with approximately 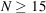 , the first directional reduction may go beyond the recursion limit of our computer; when this happens, applying first a hyperplanar shearing solves the problem. A ‘random matrix’ is a matrix whose values are randomly computed with integers between 0 and 100. Limits larger than 100, for example 1000, in large random matrices 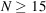 lead to too high integer values in intermediate calculations and error messages. A ‘columnar random matrix’ is here an identity matrix in which the last column is replaced by random integers in the range 0–100. Columnar random matrices are classified as random matrices and are treated with method 2.

### 4.2. Computer program and comparisons

We wrote a computer program called *Cubification* in Python 3.8 using the Numpy library to perform the matrix calculations (scalar products, matrix products, inverses *etc*.), generate the random numbers, vectors and matrices, and calculate the reduced lattices. All the results presented in the paper were obtained with a laptop computer equipped with an Intel Core i7-4600 CPU 2.1 GHz, 64-bit Windows system, with a RAM of 8 GB. The recursion limit in our Python program has been fixed to 10 000. We compared the results obtained with our program with those obtained by the LLL method computed in Python 3 by Yonashiro (2020 ▸) in a program called *OLLL*. All the *OLLL* calculations were made with α = 3/4. For specific matrices, such as that of Fig. 1 ▸, we also used the function *ReduceLattice* of *Mathematica*. On this example we checked that *OLLL* and *Mathematica* give the same result; the only difference is that the calculations are nearly instantaneous with *Mathematica*, whereas they are longer (a few seconds) with Python language (*OLLL* and *Cubification*). This shows that it is difficult to compare the time efficiency of lattice reduction algorithms with computer programs written by different people in different languages. Thus, the execution times will just be given for indication.

### 4.3. Results on non-random matrices

**Figure 4:**
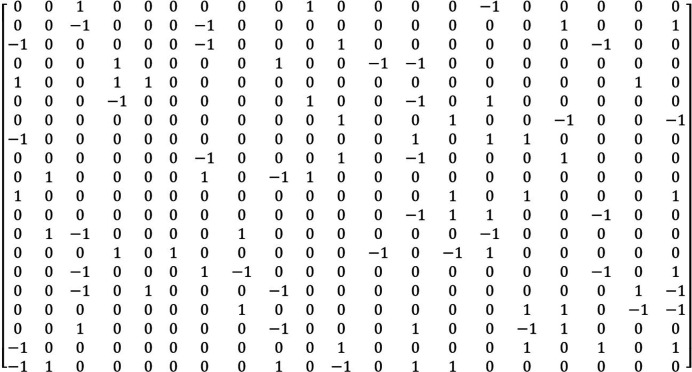
Cubification by method 1 of the lattice of Fig. 1 ▸(*a*). The vectors are written in rows, as in Fig. 1 ▸. The reduced basis has values *R* = 285, *S* = 87.

The cubification algorithm gives results quite similar to those of LLL. For example, the lattice of Fig. 1 ▸(*a*) could be reduced in three cycles (in 3.0 s); the output list of vectors is given in Fig. 4 ▸. The final basis is characterized by *R* = 285, *S* = 87; these values are lower than those obtained by LLL (*R* = 531, *S* = 99). Souvignier (2021 ▸) showed that with the Schnorr–Euchner variant of LLL it is possible to get a reduced basis with *R* = 335, *S* = 83, and then, by computing the vectors of norm 4, selecting 18 of them and associating them with two vectors of norms 3, he could obtain a reduced basis with *R* = 294, *S* = 78. These solutions are significantly better than those obtained by *Mathematica*. Compared with the result obtained by cubification, they have a lower norm *S* (also a lower norm *P*), but a larger rhombicity *R*. This example shows that improving only the norms of the vectors does not always permit a better orthogonality (and vice versa) to be obtained, as also shown in Appendix *B* [App appb].

For heterogeneous matrices, we have tested only five 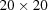 matrices, and all of them show that LLL and cubification give similar results (not shown here).

### 4.4. Results on random matrices

We have tested the performances of *Cubification* (method 2) and *OLLL* programs on columnar random matrices and full random matrices. We used matrices of dimensions 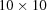 , 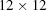 and 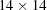 . Fifty matrices have been generated for each type. The performances on the norms and orthogonalities were measured by the reduction factors *R*(input)/*R*(output) and *S*(input)/*S*(output). The higher the reduction factors, the better the algorithm. The results are given in Table 3 ▸.

For these moderate dimensions, the reduction of the rhombicity is systematically better with *Cubification* than with the *OLLL* algorithm. The norms seem however less reduced by *Cubification* for large full random matrices. The execution times of *Cubification* are 0.1, 0.3 and 0.5 s for 10 × 10, 12 × 12 and 14 × 14 columnar random matrices, respectively, and 0.2, 0.7 and 1.3 s for 10 × 10, 12 × 12 and 14 × 14 full random matrices, respectively. They are slightly shorter than with *OLLL*. We also performed some experiments in higher dimensions. The mean execution times are 14 and 30 s for 30 × 30 columnar and full random matrices, respectively. They are shorter than with *OLLL*, but ten times longer than those reported with other optimized Python programs (Papachristoudis *et al.*, 2015 ▸). The way the algorithm is implemented, the choice of types of variables, the use of different libraries, the memory management, all play a crucial role in the execution times. In this paper, the code was not optimized to reach the best performances in execution times; its aim was only to show that simple shears along directions and hyperplanes may be interesting tools for lattice reduction.

## 5. Conclusion and perspectives

A method of lattice reduction called ‘cubification’ is proposed. It is geometrically simple; it is based on the complementary actions of directional shearing and hyperplanar shearing. These two kinds of shears were initially introduced to reduce the unit cells attached to given hyperplanes (Cayron, 2021 ▸). In contrast to LLL, the cubification algorithm does not require the calculations of Gram–Schmidt bases. The ‘driving force’ of the reduction is the ‘basis rhombicity’, a parameter that encompasses the information on the norms and angles of the basis vectors. A computer program called *Cubification* was written in Python 3.8. The results are comparable with those of LLL, at least up to moderate dimensions ( 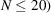 . The Python program *Cubification* is freely available from the author on request.

We foresee margins of progression for the algorithm of cubification. The two methods described in Section 4.1 were determined by trial and error; better strategies to alternate the directional and hyperplanar shears seem possible, for example by cross-calling the two processes without necessarily screening all the vectors in the basis. We could also try to generalize the 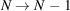 decrease of dimensions already used in the hyperplanar shearing step with the help of the left inverse matrices to work in spaces of dimensions 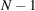 , 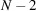 *etc*.

## Figures and Tables

**Table 1:** Two cubification methods – the values of the options are given in Table 2 ▸

Method 1	Method 2
Cubification (list, *opt.*):	Cubification (list, *opt.*):
newlist = Sort_by_norm (list)	newlist = Sort_by_norm (list)
newlist = Directional shearing (newlist, *opt.*)	newlist = Hyperplanar shearing (newlist)
newlist = Sort_by_norm (list)	newlist = Directional shearing (newlist, *opt.*)
newlist = Hyperplanar shearing (newlist)	newlist = Hyperplanar shearing (newlist)
**If** *R* (newlist) < *R* (list):	**If** *R* (newlist) < *R* (list):
**Return** Cubification (newlist, *opt.*)	**Return** Cubification (newlist, *opt.*)
**Else Return** list	**Else Return** list

**Table 2:** Method and option to be used depending on the type of square matrix We consider ‘large’ a matrix of dimension N\ge 15. For some large heterogeneous matrices a first step with hyperplanar shearing may be required before starting method 1, as indicated in parentheses.

Type of list of vectors	Cubification method	Variant for the directional reduction	
		Lagrange’s division	Simplification
Small columnar matrix	Method 1	*Insert*	*Insert*
Large columnar matrix		*Append*	*Insert*
Large heterogeneous matrix	(Hyperplanar shearing +) method 1	*Insert*	*Insert*
Random matrix	Method 2	*Append*	*Append*

**Table 3:** Reduction factors obtained on columnar and full random matrices of dimensions 10 \times 10, 12 \times 12 and 14 \times 14 by testing 50 matrices The mean deviation estimated by various tests is for *OLLL* around ± 20% for a 10 × 10 matrix and it decreases down to ±5% for a 14 × 14 matrix. It seems to be larger for *Cubification* (±25% and ±10%, respectively).

Reduction factor	*R*(input)/*R*(output)	*S*(input)/*S*(output)
Columnar random matrices 10 \times 10		
*OLLL*	2780	1000
*Cubification*	3600	1060
Columnar random matrices 12 \times 12		
*OLLL*	3120	1060
*Cubification*	4100	1090
Columnar random matrices 14 \times 14		
*OLLL*	3630	1160
*Cubification*	4370	1070
		
Full random matrices 10 \times 10		
*OLLL*	14.3	5.2
*Cubification*	16.9	5.4
Full random matrices 12 \times 12		
*OLLL*	14.1	5.0
*Cubification*	15.2	4.6
Full random matrices 14 \times 14		
*OLLL*	13.6	4.7
*Cubification*	14.3	4.1

## Appendix A Brief overview of the LLL algorithm

The most popular algorithm to tackle the lattice reduction problem was proposed nearly 40 years ago by Lenstra–Lenstra–Lovász (Lenstra *et al.*, 1982 ▸), and it is still considered as the main reference in the domain. It should be noted that the LLL algorithm does not give in general a Hermite–Minkowski reduced basis for which the vectors have minimal lengths (Ryshkov, 1976 ▸), but ‘only’ a basis made of short and nearly orthogonal vectors that constitutes a good, approximate solution that is very useful for many applications. It was initially designed to give in polynomial-time a good solution for factorizing polynomials with rational coefficients, and it is also nowadays applied for finding rational approximations to real numbers, and for solving the integer linear programming problems in fixed dimensions; it is applied in global positioning systems (GPS), data detection and communication systems. It is so important that a complete book has been devoted to it (Nguyen & Vallée, 2010 ▸). The reader can also consult Wübben *et al.* (2011 ▸). We just give here some of its key points. At the core of LLL is the Gram–Schmidt orthogonalization routine in which one attaches to any basis  an orthogonal basis  by a series of projections  =  with  . The vectors  are not integer anymore (*i.e.* ‘reticular’ in crystallographic language); they remain however rational. Practically, as the numerators and denominators may become huge numbers, floating-point numbers are used for  . The LLL algorithm works in two steps repeated iteratively. The first step is the quasi-orthogonalization. The vectors  are replaced by  , for *k* between 1 and  , where  means the nearest integer of  . The Gram–Schmidt basis should be actualized during the process. The second step relies on a criterion to determine whether or not the vectors  and  should be swapped: the swap is made when  <  , where  is a constant arbitrarily chosen between ¼ and 1 (and fixed once for all). Often, the value  is chosen. The constant α influences the strength of reduction in the algorithm and by that also the number of required iterations; greater values lead to stronger reductions; it has an effect on the final norms of the reduced vectors, and more precisely it permits the product of the squared norms  to be bound.

## Appendix B Example of a lattice with two reduced bases of the same norms but different orthogonalities

This appendix provides an example showing that minimizing the norms of the vectors of a lattice does not necessarily permit their orthogonality to be improved. Let us consider the lattice spanned by the four vectors  whose coordinates are written in rows by

The squares of the norms of the four vectors are 2, 2, 2, 3. The parameters that can be used to evaluate the ‘norm’ of the basis **B** are the sum of the squares of norms  and the products of the squares of norms  . This basis is already reduced if one considers only the norm of **B**. The output of the LLL algorithm is thus the same basis. However, the same lattice may also be given by the vectors  ,  ,  ,  , written in rows:

The squares of the norms of the four vectors are 2, 2, 2, 3, and the new basis  is characterized by the parameters  and  . The two bases **B** and **B**′ generate the same lattice and have the same ‘norm’, but their orthogonalities are different. Their metric tensors are

and

respectively. Their rhombicities are  and  , respectively. The basis  is thus more ‘orthogonal’ than the basis **B**. This example shows that the term ‘orthogonality defect’ usually attributed to the parameter *P*/*V* may not be very appropriate. Since the value  gives the Euclidean scalar products of the vectors with the other ones, the parameter  seems better adapted to characterize the ‘orthogonality defect’. The cubification method described in the paper aims at reducing both the norms and the orthogonalites of the vectors, which is why the rhombicity *R* was used as a driving force in the algorithm. The basis  of the example was found by cubification.
